# Supplementary material for: The role of external power demand on the choice of technique in classic cross-country skiing
Source: PLoS One. 2023 Mar 31;18(3):e0283794. doi: 10.1371/journal.pone.0283794 (PMC10065268; doi:10.1371/journal.pone.0283794)
Supplement: Attachment — Submitted filename: ReplyReviewerComments_clean.docx [file pone.0283794.s002.docx]

**Authors’ replies to review Comments**

Please use the space provided to explain your answers to the questions above. You may also include additional comments for the author, including concerns about dual publication, research ethics, or publication ethics. (Please upload your review as an attachment if it exceeds 20,000 characters)

- We thank the reviewers for their extensive and rigorous evaluations of our manuscript. We have considered all comments carefully, agree with most of these. Where we have a difference in opinion, we argue for our standpoint and hope that the reviewers find these in order. In these cases, the critical comments still have led to changes in the manuscript, approach, analysis and alike.
- We thank the reviewers sincerely for the many English style corrections and suggestions.
- On reference style: We see that references in the manuscript may appear awkward at places. This is due to the style used in EndNote. We have imported PLOSOne EndNote style and used it. In case references seem to appear incorrectly, we trust this will be sorted out, if accepted, in the proofing process.
- When re-running our statistical analyses, we found some settings in the Bayesian approach that needed sophistication. The outcomes have changed, the conclusions drawn were unaffected.
- We reply to all comments specifically. Many comments resulted in minor textual modifications, others in changes that were literally duplicated from suggestions. These are not specified by line numbers in this reply. Others relate to figures or reorganization of the text. Also these are not necessarily specified by line number here. For critical and larger changes, we specify line numbers in red.

**Reviewer #1: Overall Comments**
I find this manuscript interesting both as a scientist who studies both bipedal human locomotion and xc skiing. The approach and findings have implications for other scientists who study only bipedal locomotion and those who are more sports oriented. Thus, the topic seems appropriate for a general audience journal like PLOS ONE. The study design is creative and well-executed. The presentation (writing and graphs) need considerable improvements! But, that’s OK because the content is interesting and important.

I have zero expertise about Bayes statistics, maybe a statistician should take a look.

General Comments:
1. would like to see a simple diagram of the experimental set-up, just to clarify that the rope pulled backwards parallel to the treadmill surface.

We extended figure 1 and added a diagram, as well as a timeline for the entire protocol.

2. I fully understand that power is the rate of performing mechanical work. Currently, there is a mix of “work rate” and “power”. The authors should use ONE term or a shortened version throughout the paper and not mix the two. I think “external mechanical power” is the most accurate and best term to use in this paper which could and should be defined and then a shortened version could be used, e.g. “We measured average external mechanical power as the product of the summed (or total) resistive forces (gravity, rolling friction and the applied load) and the treadmill belt velocity. We use the shorter term “power” for simplicity.” Of course, the authors may prefer to use mechanical work rate throughout. In my specific comments, I have sometimes written power and sometimes kept the authors’ use of work rate just to make it clear what other changes I am actually suggesting. Overall, please pick power or work rate and stick with it.

We agree with the reviewer and have scrutinized the manuscript. We now use ‘external mechanical power’ and ‘power’ for short throughout the text. Please note that the title was changed as well.

3. The authors should consistently use EITHER “additional resistive FORCE” or “additional resistive LOAD” but not both. I would prefer force.

We agree. Text changed according (using resistive force).

4. It would be helpful if the authors noted that their method does not take into account within stride fluctuations in the kinetic energy of the COM. Perhaps they could also estimate how large/small that quantity typically is.

We have added some information, based on our data set. L439-443

5. I suggest that the flow of the Introduction could be improved, especially for a non-specialist readership. I would suggest that the order should begin with the familiar (human walk-run transition), touch on quadrupedal gait transitions and then onto the less familiar classic Nordic skiing sub-technique transitions.

When preparing the manuscript, we have discussed this issue in general terms and came to the current solution (by ‘matter of taste’). We do however see the reviewer’s point and have changed the order accordingly: moving content of lines 60-62 to the first paragraph.

6. Again, try to use just one term “trigger” rather than sometimes trigger and sometimes “controlling factor/parameter etc.” I prefer trigger.

We now use ‘trigger’ throughout but explaining once that this is the same as ‘control parameter’ in the dynamic system approach literature on motor control. Furthermore, hysteresis is also introduced early (L38-39).

FIGURES
Figure 1 needs a label for horizontal time axis units, I believe they are seconds.

Correct, now fixed.

Figure 2 Colors {Grey/Green for DP, Green/Purple for DPK and Purple/Grey for DS} are which are not the same as Figure 1 {green (DP), blue (DPK), and red (DS)}. Please make the colors more consistent. What is meant by grey?
Figure 3 & 4, then use green and blue but to indicate incline!? Either use different colors for incline or just add 5% and 8% text inside or near the boxes. Further, speed (velocity?) is now in m/sec but in Methods section etc. 10 & 12 km/hr. Be consistent!
Figure 5 uses grey but not with the same meaning as in Figure 2.
We have changed the color signature in the figures (amongst other important changes). The color signatures for Figs 1-3 are now corresponding. Figs 4-5 (force/power at transition) have no signature relationship with Figs 1-3 (aspects of techniques), thus, to avoid confusion, we use no color signature in Figs. 4-5

We also now only use one unit for speed throughout (km/h).

Table 1 caption: outcomes (plural).
Corrected

Minor Stylistic Comments
TITLE:
PLOS ONE is a non-specialist journal. I am an avid Nordic skier and biomechanist but probably, more than 99% of the international readers have never roller skied or even heard of roller skis. I’d bet that with the current title, most PLOS ONE readers would guess that your research is about in-line roller skating. Probably <10% have ever enjoyed nordic skiing. But, many have seen/watched nordic skiing on television once every 4 years but most know it as “cross-country skiing”.
I encourage the authors to just use “classic cross-country skiing” in the title.
I understand that in the sport-specific Nordic skiing literature, the terminology is usually two “techniques” (classic and freestyle/skating) and then “sub-techniques” within each technique. However, in the title, I like how the authors just use “technique” because “sub-technique” would require an explanation to a general reader. As I discuss below, the authors should decide if they want to use work rate or work-rate or power or external mechanical power etc. etc. and then use that term in the title. I also suggest that the authors simplify the body of the paper by just using “technique” throughout.
We agree with the reviewer and have changed the title according all these suggestions. We just wish to note that previously we have been criticized using ‘cross-country skiing’ instead of the more specific ‘roller skiing’. Yet, in the context of this study, the difference is hardly of relevance, thereby the original wording confusing.


ABSTRACT:
Here and throughout the manuscript: decide if you want to use “velocity” (v) or “speed” and then use it consistently, don’t switch back and forth.
We use speed throughout now, with 2 contextual exceptions where “velocity” seems the best term to use.

Line 13 Add comma after cross-country skiing, Done

L13 suggestion: …use different techniques akin to locomotor gaits such as walking and running. (I don’t think the “gear” analogy is very effective on its own and requires an explanation).
We agree, changed. Please note that the skiing literature mostly refers to ‘gear’.

L14 what do you mean by “demand”? why not just say “techniques generally depend on speed and incline.”
Changed

L15 roles (plural)
L15 “… roles of speed, incline and mechanical power demand in triggering technique transitions.”
Adopted

L16 cut sentence beginning: However…
L17 change this sentence to “However, it is still not known if mechanical power demand, as an isolated factor, has any role on the choice of technique.
Adopted

L19 …choice of technique during classic cross-country roller skiing by changing mechanical power demand at fixed speeds and inclines.”
Adopted

L21 … at the four combinations of two speeds (10 and 12 km/hr) and two inclines (5 and 8%) while additional resistive forces were applied via a weight-pulley system.
Adopted

L23 Athletes were free to choose between three techniques (double poling, double poling with kick….
Adopted

L26 Obviously power is affected by both incline and speed, but the focus of this paper is the trigger(s) for technique transition. Thus, I suggest the overall findings be summarized as: “At a given incline, technique transitions occurred at similar additional resistive force magnitudes at the two speeds but on the steeper incline, the transitions occurred at smaller resistive forces. Transitions were not triggered at similar mechanical power demands across the different incline/speed/resistive force conditions. This suggests that mechanical power itself is not a key technique transition trigger.”
Thank for this nice suggestion. Adjusted accordingly.

L30 “but only when incline is not involved” is very confusing.
Changed

L31 “…suggest that technique transitions in classic cross-country skiing are not triggered by a single factor.”
Adopted

INTRODUCTION
L36 These phenomena are well-studied Adopted
L39 Yet, while minimizing metabolic cost… Adopted
L42 …challenge exists for understanding the transitions… Adopted
L42 I suggest that you don’t worry about skating, and cut the gear analogy. just write: In classic-style XCS, skiers make transitions between three main techniques: double poling (DP), …. Adopted
L46 cut “the heaviest gear” Adopted
L46 with propulsion generated… Adopted
L47 DP is usually chosen for flat/low angle terrain when speed is fast. Adopted
L48 cut “lightest gear” Adopted
L49 …leg movements, with both poles and skis transmitting propulsive forces. Adopted
L50 DS is usually chosen on uphill terrain when speeds are slow. Adopted
L51 cut gear Adopted
L51 similar to DP but with the addition of one ski thrust per poling cycle. Adopted
L53 more influential parameter (9,10). Adopted
L54 …are tightly coupled. Average external mechanical power is equal to the product of velocity and the sum of the resistive forces (i.e. gravity, friction and aerodynamic drag forces). Thus, even though techniques chosen are generally linked to incline, it is not apparent if one factor (incline, speed, or power) actually triggers the spontaneous transitions between techniques. Adopted
L58 cut sentence “Even less so…” Adopted
L60 move this walk-run sentence earlier in the Introduction, following my overall ordering suggestion. Adopted
L65 on steep uphill terrain and at slow speeds. Adopted
L65 This idea was challenged by Dahl & Sandbakk (12) who suggested that on steep uphills, the mechanism…. Adopted
L67 specify: utilization the body’s gravitational potential energy Adopted
L70 on steep inclines Adopted
L72 at fast speeds Adopted

L73 on flat terrain (11,12). But, Ettema & Kveli (9) disagree and suggest that…. briefly explain their argument here.
We added extra information L75-77

L75 cut first sentence and replace with the following suggestion.
L76 Two main study design approaches have been used to try and disentangle the problem of technique transition triggers in XCS. Adopted
L78 …different but prolonged conditions allowing athletes to choose or indicate the technique they prefer. Another approach has been… Adopted
L81 This acute approach seems better for quantifying… Adopted
L84 our research group tried to elucidate… Adopted
L85 triggering factor Adopted
L86 predominant trigger. Adopted
L90 the evident limitation of Ettema & Kveli is that the role… Reference inserted.
L91 provide names for (10), e.g. Lokkeberg & Ettema circumvented this… Adopted
L91 by using an extended protocol… inserted
L93 only partly supported the idea that incline is the dominant factor. Adopted
L93 Lokkeberg & Ettema concluded that work rate alone played… Adopted
L94-97 I don’t understand this sentence/study and can’t help you to make it more clear.

We rephrased. The rationale is that if power truly plays a key role, one would not expect similar use of techniques at this submaximal intensity as well as during competition intensity L95-98

L98 start a new paragraph for hysteresis and first define what it means. Adopted. Hysteresis is now defined/described early in the introduction (for walk-run). We have a brief (likely somewhat obscure) description here as well – which can be removed.
L104 …protocol that changed work rate at fixed speeds and inclines. Adopted
L108 work rate was changed at fixed speeds and inclines. Adopted
L108 This was achieved by applying different backwards pulling forces (parallel to the treadmill surface) while the athlete roller skied at fixed speeds and inclines. Adopted, with slight differences. The force was approximately parallel to treadmill surface. L109-

L110 The comparison of four such conditions (2 speeds x 2 inclines) allowed us to distinguish the specific effect of work rate from the speed/incline effects. Adopted
L112 Our overall thinking was that if athletes transition between techniques at the same…. Adopted
L113 then, restate this thinking as a Hypothesis. We hypothesized that at different fixed speed and incline combinations, technique transitions would occur at the same work rate. Adopted

METHODS
L119 it sounds like some subjects were dedicated skiers whereas others might be footballers, gymnasts, swimmers??? Indeed, we should have clarified better. ‘their sport’ refers to XCS, now specified. All were member of the university student ski team. L122
L127 warm-up is hyphenated Adopted
L132 some backwards pulling resistance force was added… Adopted
L133 ones might have exceeded some individuals’ anaerobic thresholds. Subjects’ anaerobic thresholds were not measured... Adopted
L137 The four combinations… Adopted
L138 were studied. (note: don’t overuse “applied”. I suggest: The resistive force was “applied”, the technique were “chosen” and the conditions were “studied”. Adopted
L140 would not choose DS technique at the onset of the protocol because DS is usually chosen on steeper inclines. Adopted
L142 between the athlete and a hanging weight. Using a broad belt… Adopted
L145 specify that the height of the pulley was adjusted so that the pulling force was applied parallel to the treadmill surface. (p.s. good idea to use a bicycle wheel! Very low frictional torque at the rim.)
We have now specified. However, we wish to be honest, indicating that this was done by approximation. L151-153
L145 The weights consisted of small bags… Adopted
L148 separate sentence: A 500g hanging weight corresponded to a ~13-16 W change in external mechanical power. Adopted – in slightly modified way L155-156
L149 at the same time Adopted
L150 if this DS technique was maintained Adopted
L150 Occasionally, on the 5% incline, an athlete did not choose the DS technique even after adding considerable resistance. In such cases, the bout was terminated. Adopted
L161 …own poles or choose poles provided … intervals of 5 cm. Adopted
L173 I suggest just using the one term “trigger” and not add another term for the same thing (control parameter) We have scrutinized the manuscript for use of terms. We use ‘trigger’ all out.

L174 Here is a good place to define formally either “external mechanical power (or work rate)”. Done
L174 it is also a good place to mention that other work is done (internal limb work, COM KE fluctuation work) Adopted in slightly modified manner. L190-193

L177 probably should use “velocity” throughout the paper if you want to use v (which is fine). Should also specify that this v is the treadmill belt velocity. We agree that ‘speed’ may be the correct term in the context used (as is done in the literature). Still ‘v’ is the usual symbol to use in equations. We stick to speed in text, explain the use of the symbol ‘v’ in its most simple way. L181-182
Manuscript is now scrutinized for term use.

L178 rolling friction Adopted
L180 allowing us to distinguish (and cut: as such) Adopted

L181 external resistive force, or external impeding force or whatever you decide to use consistently

We now use ‘(external) resistive force’ consequently. We added also total resistive force (reviewer#3), thus we now make the distinction between ‘additional’ and ‘total’.

L181 I’m confused here. DS involves two propulsion and glide phases per stride cycle. I suppose you could say “Because all classic xcs techniques include at least one propulsion and one glide phase per stride cycle, the external resistive force fluctuated … Adopted

L193 The main statistical tests were comparisons between the external resistance forces and mechanical powers at the technique transitions across the two incline and two velocity conditions.
Adopted

L196 any difference in the external resistive force at the technique transitions or mechanical power at the technique transitions between the four incline-speed conditions is regarded as evidence that the variable is not an isolated trigger factor. In some cases, an athlete did not choose all three techniques (i.e. they did not choose DP or DS) in all four conditions. Adopted

L199 I really do not understand the sentence beginning: In such cases…

We modified this sentence. The main idea is that if an athlete did not use, for example, DP in one incline-velocity combination, but did so in the others, a difference in the DP-DK transition is present, i.e., no transition occurs versus a transition occurs at a given force/power. Thus, a comparison can be made, but the data set lacks a number (force/power), i.e., the statistical analysis treats it as missing value. To avoid this, a fictitious number was introduced, a force or power just outside the range we recorded. The exact value has no or little impact on the statistical outcome. L215-220

L205 I agree you are testing a null hypothesis, but you should have stated the Hypotheses way back in the Introduction. We agree, this is now changed in the introduction. L114-116

L208 To test for a technique transition hysteresis, the external resistive forces at the technique transitions were compared between when the force was being incremented vs. decremented. Adopted
L209 no need to (Note...) parentheses. Changed

RESULTS
L216 …powers for which each technique was preferred. We changed but replaced “preferred” by “chosen”.

L222 powers at transition differed This is incorrect; we refer to mean powers while these techniques were used. We rephrased to avoid misunderstanding. L247-248
L227 Time using DS … time using DP decreased Adopted
L228 Speed had no effect (on what?) Now specified: on time used… L253
L229 time using DK was not significantly affected by incline: …0.308 or velocity: p=0.296… Adopted
L242 specify how many athletes
L242 X athletes did not choose to transition between all three techniques in all four conditions. Adopted, slightly modified, numbers are now included L269-270

L248 outcomes of the ANOVA are Adopted
L249 Results section should all be in past tense: were affected Adopted
L249 explain what you mean by reversed fashion We expanded, explanation now Included L279-280
L250 Power at technique transitions were also affected by velocity which was not the case or resistive external force… Adopted
L261 explain what a positive hysteresis means in words, i.e. something like: subjects retained a technique at greater forces when resistance was being incremented vs. decremented. Included

DISCUSSION
I usually write the Discussion in past tense while reviewing/summarizing the results. Adopted
L269 the independent role of mechanical power on technique transitions in classic cross country skiing. Adopted
L271 The findings confirm the general expectations regarding technique preference: DP was chosen at the lower power output range, DK at medium powers and DS was chosen at higher powers. Adopted
L273 ranges was Adopted
L274 mixing your terms here: load = ? terrain = ? I don’t understand this sentence. We changed terrain into ”incline, velocity, snow conditions”; load is replaced by power
L275 Athletes tend to choose the DS technique more on steeper inclines and DP on shallower inclines. Adopted
L279 Both power at transition and resistance force at transition were strongly affected… Adopted, slightly revised
L281 cut (control parameter) Adopted
L282 should be noted that we tested the null hypotheses that technique transitions would occur at similar resistive forces and power outputs regardless of incline or velocity. The challenge of this approach… Adopted
L285 any factor (resistive force or power output at transitions) showing significant differences between conditions is not a trigger, but the other factor might be. Adopted
L289 only velocity was Adopted
Page 14-15 -16 is a VERY LONG paragraph! Break it into several paragraphs each with a topic sentence. We made an attempt. We have now smaller paragraphs and changes some of the ‘entrance’ sentences.
L297 studies. Both Ettema & Kveli…. Adopted
L300 This is the MAIN FINDING but it is buried in the middle of this gigantic paragraph. Try to give it more attention, probably as the topic sentence of a shorter paragraph. Using smaller paragraphs, this main message is now contained in one such paragraph (10 lines). We added subtopic titles.
L302 cut: “In the current study” Adopted
L304 (9,10), here mechanical power was manipulated independent of velocity and incline. Adopted
L304 Still, the most likely conclusion supports our previous findings: rather than mechanical power output itself, one or more of the underlying factors (e.g. external resistance) may be a trigger for technique transitions. Adopted
L308 were chosen despite the fact that aerobic intensity was only moderate. Further, in xcs competitions, when aerobic intensity is clearly very high, all three techniques are chosen under different conditions (terrain, snow friction, wind etc.). Adopted, yet we did not insert ‘aerobic’. We do not regard the energy source (aerobic vs anaerobic) an issue in this case, at least not one we can conclude on. L361-363
L311+ Even though people tend to use the gait (walking vs. running) that is energetically most economical, energetics are not a direct trigger to transition between gaits. The change in movement economy (i.e. VO2) changes too slowly to be considered a trigger for an abrupt gait transition (see 16). Other variables that do change more abruptly (i.e. muscular factors) are more likely triggers. Adopted in slightly modified manner L365-371

L317 Again, this is a very long paragraph. I suggest breaking into multiple paras This paragraph has its own sub-title and now has two paragraphs, one of which is still long, unfortunately.
L318 transitions between speeds at the same incline, there were considerable interindividual differences. Adopted, we added “but” “…, but there were …”
L319 differed by only Adopted
L320 at the two treadmill velocities. Adopted
L321 Therefore, external resistance force may play a trigger role on a fixed incline. Adopted
L324 …trigger role’). Yet, the difference… Adopted
L327 effective at shallower inclines Adopted
L329 is the intrinsic trigger. Adopted
L332 force is the corresponding internal trigger candidate. Adopted
L336 athletes changed Adopted
L337 to DS with increasing demand Adopted
L339 cut (and as mentioned above) Adopted
L340 …mechanical energy for propulsion via the legs becomes less effective... We rephrased, but in DP propulsion does not go via the legs. L399-401
L342 This leads to the choice of DS because the legs can generate considerably more power… Adopted
L344 other mechanism contributes to triggering the transition to DS on steep inclines. We cannot.. Adopted
L346 key role (11). Cut rest of sentence Adopted
L348 extremities can contribute Adopted
L351 …and by transitioning to DS, the propulsive force can be distributed between both poles (arms) and skis (legs). Adopted
L352 Thus, while we previously found evidence against a pole force trigger (12),… Adopted
L356 likely has Adopted
L359 gait transitions. Adopted
L360 Curiously, one of the athletes… Adopted
L367 was outside of their individual range…. Adopted
L367 was there anything notable about these athletes in terms of skiing experience/expertise? There was nothing notable – this is now noted in the ms. L455-456
L375 …conditions. Like in the walk-run gait transition, hysteresis is an inherent Adopted

L381 external resistance force Adopted
L383 cut Rather Adopted
L384 more precise, but not essentially different. The outcome of this study…. Adopted
L390 plural: fluctuations Adopted
L393 “not generated” is confusing. Sentence rephrased
L396 I suggest a final summary paragraph. We moved the last 2 paragraphs of the long section in the original version to the end, which now acts as concluding-summary paragraph.

**Reviewer #2: Overall Comments**
I believe that the authors’ research focus and the data originality are within the PLoS policy. The introduction section was well written and logical study necessity and hypothesis can be found except for some minor mistakes (P3 L50 … choice ‘of’ and … and P3 L66 … still very much… etc.). English and/or sentence editing is highly necessary even if you are native English speakers. However, probably due to a lack of appropriate number of participant, statistics and result sections are confusing.

Major comments
1 P3 L43. You need to present a sample movie to explain DP, DK, and DS.
Thank you for this suggestion. At this stage of the revision process we have not added a sample movie in the supporting material. Should the reviewer and editor insist this is a necessary step, we are happy to make a movie that shows the specifics of these three techniques.

2 P5 L123-L124. You should show the production name of the used treadmill and classic roller skiing at the first appearance, but not in P6 L151 and L153.

We re-ordered some of this information. The information in the last par before “Recording Equipment” has been moved upward.

3 P5 L122-L135. You showed a typical example in Fig. 1, but explanations of a series of the protocols should be presented as a figure before presenting the current Fig. 1.

We extended figure 1, now including the setup on treadmill and pulley force, as well as a timeline of the entire protocol.

4. I seriously confused why the authors mixed to use Bayesian analysis, box plots, and 2×2 repeated measures of ANOVA without checking data normality for this small sample size. I hope you to analyze with usual statistics in biomechanics or sports physiology area. The result section and Figure presentations should be conducted from the scratch. Bayesian approach has been used for estimation of available parameters, future prediction of big data, choice of the mathematical model, and its statistical analysis. Even if your statistics is correctly used, more participants could be necessary.

We agree with the reviewer that we might have been confusing readers by combining parametric statistical tests and box plot-figures, which are non-parametric in nature. Thus, we have changed all the figures to correspond closer to the statistical tests performed. In the figures, we now show all individual values as well as mean±SD.

In our opinion, while we appreciate that the frequentist approach is by far the most used (still), restricting analysis to the “usual” is no valid argument. Bayesian inference can be used for anything, it is just another way of making inference. In this study, we choose to present both frequentist and Bayesian inference, both because the two methods have their strengths and because we appreciate that the general reader still is most used to frequentist p-values. We agree that in general one quickly relies too much on p-values above or below some threshold (e.g., 0.05). Including Bayes factor (as well as Bayesian estimated mean differences including spread in the form of 95% credible intervals) together with p-values, we have a more holistic approach (rather than solely focusing on ‘significance’) thus reduce the chance of committing type I errors.

We have extended the statistical analysis reporting (Table 1) with F values and effect size (eta2).

Regarding low N, for all models the residuals were checked for being approximately normally distributed, which they were. Now mentioned in text L229-231. Furthermore, while we agree our sample size is not very large, we do not fully understand the comment about low statistical power, since we have highly significant effects on many factors (power approaching 1). With a low N and low p-value, we obviously have large effect sizes and thus very large statistical power for these variables. We humbly disagree with the notion that Bayesian statistics can only be used for large N. We now use several references that is more appropriate for our application of this approach. L220-229

4-1. The authors need to justify the estimation of the original sample size. In my calculation (effect size = 0.25, alpha level = 0.05, statistical power = 0.8, if correlations among repeated measures are high enough = 0.7-0.8), you need to test at least 16-18 participants.

We agree in principle with the reviewer, and our original sample size was 14. See also answer to the above comment. We argue that the more ‘holistic’ approach of and use of both Bayesian (directly comparing relative probabilities of H0 to H1) as well as classical methods – while it does not inherently allow for a lower number of participants – it helps in reducing the chance of type I and II error. Note that we do not overemphasize whether p-values (or Bayes factors) are below or above some threshold, rather we look at the data and statistical outcomes as a whole.

We do not agree that our data set leads to too low statistical power. We wish to note that, for many main outcomes, the actual statistical power approaches 1. For example, power at transition DP-DK differs with ~20W +-SD 5.7. At <0.001 alpha this gives a power close to 1. We argue that, if our outcome parameters (e.g., force and/or power at transition) tend to differ at transition points, our sample size and study can detect such indications given by the data, especially when compared to parameters that tend to not differ (pulley force, or total force, for the DP-DK transition). Example wise, if an actual true difference (paired, two-sample t-test) would be 0.8-+SD=~12 (effect size = 0.1), our statistical power is <1% with N=11, and we would indeed need N>700 to detect this as a statistical difference (p<0.05) at a power of 80%. This difference would in our study still be totally meaningless, which would be supported by a Bayes factor close to 1.

4-2. The effect size (η2 values for ANOVA and Cohen’s d values for t-test) and degree of freedom need to be provided in this small sample size. Did you confirm the data normality before performing ANOVA? How were the F values? T-test (as a post hoc test in this study) also requires data normality. Note that the effect size and p-values are dependent on the degree of freedom.

For the key analysis, we have included effect size (eta2) and F-values in table 1. For readability of the results text, we refrain from presenting all statistical information at all places, which we argue often is of technical character. Rather, we present the n-number which is more informative for the general reader (of course this is open for change if the reviewer/editor insists). Please note that some ANOVA’s and the t-tests regard analysis of descriptive nature (presenting the dataset about use of techniques), not the core of the study.

4-3. Even though you tested small number of participants, you used 2-way repeated ANOVA before checking the data normality. In addition, why did you combine Bayesian approach and ANOVA?
We refer to our earlier reply (4 and 4-1). The normality check is now inserted in the text. L229-231

4-4. There is a serious claim for presenting box plots in Figs. 3-5. DO NOT use the box plot in your case because it sometimes provides the same plot even if the data distribution is completely different. Herein, the problem came from the ref. #15. The #15 seemed to aim for a large sample size (200-5000) for PSYCHOLOGISTS. You used biomechanical technique, so I ask you whether it is appropriate for your study design (I do not think it is appropriate). Usual bar presentations are necessary instead of box plots.

We refer to our answers on previous comments related to statistics. We have changed the presentation form of our data in these figures accordingly.
Our reference #15 was in a way misleading regarding sample-size, however the argument for the default prior is in principle independent of sample size. We disagree, however, with the notion that Bayesian analysis can only be used for large samples.

5. P10 L260. In your statements in the introduction section, the aim of this study was to examine the role of “incline and speed”, but not “role of work rate”, wasn’t it? Check in detail.

6. English editing is highly necessary in the discussion section. It sometimes lacks subject, and too many spoken English apparently existed (e.g., P10 L275 … is likely not …; P10 L280 and L281 at ″the″ same…; P10 L287 a bigger role… should be rephrased ″… more important role in …″; P11 L307 …different speed, be it with… sentence looks strange.; P13 L367 Like in our previous studies… Being similar to the results of our previous studies [refs.]…)

We appreciate that our English is not perfect. We checked these specific comments. Moreover, reviewer#1 has made an abundant list of English corrections, which we adhered to. We hope that this suffices.

7. Summarizing above, the whole discussion, results, and statistical sections should be re-written. At least, I do not agree with this interpretation that is not linked with your own results. Indeed, observed results (figures) were not referenced in the second, third, and fourth paragraphs of the discussion section.

In accordance with comments by this and the other reviewers, sections have been modified considerably, including use of sub-sections and paragraphs moved elsewhere. Still, the main conclusion is not altered but some of our interpretations and conclusions have been modified somewhat during this revision process, yet not changed in essence. We respectfully disagree with the idea that these sections in the discussion are not based on our findings. Where appropriate, in these paragraphs, we now explicitly refer to tables and/or figures.


8. Why does the DP, DS, and DK overlap at wide range of sped and incline? Other factors that the authors ignored could be related to the observed phenomenon, such as metabolic and muscular influences.

We do not understand this remark very well. In our opinion, the range of speed and incline is actually rather narrow but includes speeds and inclines where athletes tend to shift. In the discussion, we debate metabolic and muscular factors, which are interlinked with intensity and choice of technique; such factors are not independent of each other. We did not ignore these factors but took external conditions as departure point, after which we discuss which ‘internal’ factors (metabolic, muscular, sensory) could play a role. See for example Ref#Marshland on overlap in speed (under actual ski racing) for all three classical sub-techniques, this ref now included in text. L312-313

9. Six of 19 references (more than 30%) are based on your own studies. It looks turning everything to your own advantage. Background check could be lacking.

Indeed, we refer to our own work to great extent. However, the reference list entails – to our knowledge – about all studies of relevance on technique choice in cross country skiing (a list that is not extensive). In our opinion the ‘advantage’ comment may be fair but, indeed, it only ‘looks’ like that. At least, and we hope we are trusted on this, we never intended to merely ‘proving our point of view’ like a self-fulfilling prophecy. In fact, we retract one of our conclusions from a previous study, and support others’ (Pellegrini). We have added some more literature other than our own regarding technique mechanisms and preference depending in incline and speed. One more reference of our own group (Solli et al) is now used to show self-criticism – we dispute their statement that speed determines technique choice.

**Reviewer #3: Synopsis:**
This study aimed to isolate the effect of work rate on sub technique selection during classical style roller skiing by varying resistance force using a pulley system attached to the participants pelvis. This enabled variation of resistance force and work rate at constant speed and incline. The results show that work rate at self-selected transition between sub-technique was dependent on both speed and incline, and hence is not a direct trigger for transition. The pulley force at transition was independent of speed, but dependent on incline. Therefore, the authors conclude that resistance force might be a direct trigger for transition when incline is constant.

Assessment:
The study has a simple but clever design that apparently allows to distinguish the roles of incline, speed and work rate. It clearly adds new insight to previous studies on the same topic. However, if I interpret the results correctly, I believe the authors have overlooked the effect of gravity, which also works as a “resistance force”, when interpreting their results.

Major comment:
An important question that I think the authors do not discuss in sufficient dept is: does it matter by which mechanism resistance force is increased (e.g. by increasing incline or by adding string force close to the participant’s center of mass)? If we assume that the mechanism resistance force is added by is of minor consequence, it seems obvious that “resistance force” should be seen as the sum of string force, gravitational component along treadmill, and rolling resistance. Since the latter was constant in this experiment, and is relatively independent of incline, it can be neglected. However, the gravitational component obviously changes between the 5% and 8% incline conditions. By my calculations, the increase in resistance force due to gravity between the two inclines will be about 20 N (69 kg * 9.81 m/s^2 * [sin 4.57° - sin 2.86°]). If this is accounted for in the ANOVA (Table 1), it seems it will more than compensate the difference in resistance force that the authors report (6.7 N and 2.9 N for DP-DK and DK-DS, respectively). Based on that, I encourage the authors to reconsider how they interpret their results, and possibly modify their analysis to accommodate the change in gravitational resistance force.

We thank the reviewer for this insight. We have included total external resistance (pulley, gravity, roller friction) as a third variable for analysis. Indeed, some of the findings on pulley resistance and total resistance do not match. After some thought, we realized that at equal speed, when only regarding incline effect, the total resistance force outcome resembles that of external power since P=Ftot*v. Interindividual differences in power and force amplitudes in the outcome are explained by body mass (Fig 4 top and bottom diagram show identical trends, interindividual amplitudes differ). At equal inclines, only regarding speed effects, the analysis on string resistance and total resistance are almost identical (the minute statistical differences are likely due to that body mass variation causes a change in total variance compared to string force only). We explain this in the discussion. L2778286; 318-325; 391-394

Minor comments with line numbers:
- Line 14: “demand” is not very specific, consider rewording for clarity Modified (reviewer#1)
- Line 24 and throughout: “power” and “work rate” are used interchangeably. Consider standardizing. Modified (see also reviewer#1)
- Line 55: “one of these is the product of the two remaining” – this is not strictly true, suggest to either clarify or remove. We agree that strictly, the formulation is incorrect. We simply removed
- Line 68: Missing numeric reference after Danielsen et al. Inserted
- Line 84: “In a few papers, …” Sounds very colloquial, consider rewording for conciseness. Changed
- Line 88-90: Consider omitting the parenthesis. Check that the reference is formatted correctly. Changed
- Line 112: use sub-technique (or gear, if you prefer) instead of “technique” for improved consistency We now use ‘technique’
- Lines 117-118: Subject heights are reported using incorrect units (even Norwegians or Dutch aren’t that tall…) Our sincere apologies for such terrible mistake, corrected
- Line 133-135: consider rewording to simplify the message. How is it relevant that the subjects were familiar with the term “anaerobic threshold”? We agree. We used this term explaining to the athletes about what they should expect. But indeed, not relevant to mention in the manuscript. Second sentence in parentheses is removed. L143
- Line 179-181: Please consider this in light of my “major comment” We have included total resistance force as variable. Here we introduce the reason (different relative pulley weight because of body mass differences). In the discussion, we explain how total force and power give the same outcome at same speed. Further, it is discussed when deliberating propulsion. L185-190
- Line 189: Consider specifying which condition these data are from (speed and incline) We have now included this info.
- Line 220: should it really be p=0.001, or is it p<0.001? We checked the files. The value was indeed =0.001 (not <)
- Table 1: include units where relevant (for force and power) Adopted
- Line 347: “However, the poling force idea makes sense”. Consider rewording, very colloquial. We rephrased L406-407
- Line 396: It might not be mandatory, but I’m missing a conclusion at the very end of the manuscript. We added a short summary/conclusion paragraph/sub-section. We moved a part of the discussion to the end, now under its own heading (see reviewer#1)
