# Supplementary material for: The role of external power demand on the choice of technique in classic cross-country skiing
Source: PLoS One. 2023 Mar 31;18(3):e0283794. doi: 10.1371/journal.pone.0283794 (PMC10065268; doi:10.1371/journal.pone.0283794)
Supplement: Attachment — Submitted filename: ReplyToComments02.docx [file pone.0283794.s003.docx]

**Authors’ replies to review Comments #02**

We thank the reviewers once more for an elaborate evaluation. We reply here only explicitly to those questions that require a more extensive answer than simply “amended” or “adopted”. Those questions are indicated in red - our reply in blue. All other comments are adopted literally.

We have once scrutinized the manuscript for ‘sub-technique’, which are all changed into ‘technique’.

Please use the space provided to explain your answers to the questions above. You may also include additional comments for the author, including concerns about dual publication, research ethics, or publication ethics. (Please upload your review as an attachment if it exceeds 20,000 characters)

Reviewer #1: The authors have been very responsive to my suggestions for improving the clarity of the writing. I have no substantial issues, only minor grammatical/clarity suggestions

Title Consider adding “demand” The role of external power demand on the choice…..

Abstract
Line 24 get rid of the parentheses - …three techniques: double poling, double poling with kick and diagonal stride.
Line 28 Importantly, technique transitions were not ….
Line 32 …power) may be transition triggers when incline is fixed and speed is changed.
Line 41-42 Yet, while minimizing metabolic cost…
Line 46 …understanding the technique transitions in cross-country skiing
Line 51 DP is usually chosen on what is regarded as easy terrain, where speeds tend to be fast.
Line 66 on steep uphill terrain and at slow speeds.
Line 72 steep inclines and at slower…
Line 74 …[16,17]. However, this has been…
Line 76 …at different speeds. If one transition …
Line 76 … another one must have been at slower or faster speeds.
Line 77 suggesting that a unique…
Line 79 One approach has involved prolonged conditions…
Line 81 and record the conditions at which athletes change techniques spontaneously.
Line 96 …is supported by the finding that both at extreme …
Line 98 are chosen [19].
Line 103 … power in influencing the choice…
Line 108 cut “sub-“
Line 113 if athletes change techniques…
Line 134 provided by the laboratory
Line 137 During a ten-minute
Lines 138 & 140 cut “sub-“
Line 144 each bout (i.e. one speed-incline…

Line 145 km/hr or km hr-1 We added the space but prefer ‘h’ instead of ‘hr’, both of which seem to be accepted in the literature. Figures and some other places the same correction was made.

Line 146 were selected based
Line 147 expectation (singular, cut the s)
Line 158 In such case, one… (add comma)
Line 172 a custom algorithm [15]
Line 177 potential triggers (add “s”)
Line 178 … directly, the external mechanical power…
Line 179 and adding the power required to overcome the resistive force (Fp)
Line 188 cut “also”
Line 190 was also investigated as a possible trigger.
Line 190 only the mean

Line 194 during each stride cycle

Line 203 I don’t like “pulley weights” since the weight of the pulley itself is not relevant. I suggest: …showing hanging weights. C. Cycle…. We have changed accordingly in the Fig.1 legend, with slight modification.

Line 211 cut: “at the technique transitions”, you repeat it later in the same sentence.
Line 215 all four conditions.

Line 216 … as ‘missing value’. Yet, non-transitions can also provide valuable information. Cut: “as those cases where a transition did occur” Amended, but also in accord with other reviewers’ comments. L.219-222

Line 232 cut “a”
Line 269 cut “sub-“
Line 269-270 all four conditions
Line 276 …combined transition data for increasing…
Line 279 in an opposite fashion;
Line 284 … at transition were affected by incline in opposite ways.
Line 285 … at transition were nearly identical. (not merely! ϑ)
Line 313 This suggests that the factors that determine external power (i.e. incline, snow conditions, speed) are all important for the choice…
Line 314 cut “sub-“
Line 315 cut “sub-“
Line 315 at the steeper inclines, …
Line 316 at the shallow inclines
Line 316 equally as much
Line 319 … by incline but opposite manners, while minimally affected by speed.
Line 321 at fixed inclines, (plural)

Line 324 despite the fact that gravitational… - changed to ‘even though’ (“Word” suggestion)

Line 325 at transition) comprised at least..
Line 338 is not. Comparing transitions at the same…
Line 339 at the same reistance, not the same power
Line 340 values for the speed effect on the force at transition, it should be noted…
Line 343 we cannot conclude that resistive
Line 344 where only incline was changed neither
Line 345 with the exception
Line 353 + cut: “also when not determined by speed or incline”
Line 354… be regarded as a trigger for technique transitions.
Line 355 External power is calculated from a combination of speed….
Line 360 cut “sub-“ twice!
Line 364 external power itself unlikely…
Line 364 trigger for technique transitions…
Line 371 likely triggers, though not easily measured.
Line 380 vs.
Line 389 start a new paragraph at: Moreover, we cannot…
Line 394 start a new paragraph at The current findings…
Line 398 athletes did the same
Line 401… mechanical energy, i.e. “falling on the poles”.
Line 408 all propulsive force ultimately is applied via the poles
Line 409 At the higher resistances
Line 411 become too great.

Line 413 paradigm – supports the pole force trigger hypothesis. Adopted, but slightly modified (‘to some extent’), our data do not suggest that resistance force definitively is a trigger. L 426

Line 429 we have no evidence
Line 439 not caused them.
Line 439 inherent to XC skiing.

Line 440 in this study, (add comma)
Line 449 I suggest adding: “However, based on the present study, we can now reject external mechanical work demand as an important trigger for cross-country skiing technique transitions. We replaced the sentence with this suggested one (slightly modified). L487-489

Line 451 re: (visual) it might be interesting to explore if visual optic flow affects xc ski technique transitions during treadmill roller skiing. It seems to be a hot topic now according to Google Scholar. Google: “optic flow gait transition”

We agree, thank you for the tip. Effects of optic flow in general has been a traditional topic in motor behaviour. In the manuscript, we added a reference [33] on this issue.

Reviewer #2: The manuscript was greatly updated. Most of them are good, but there are several additional requests for the authors.

1. L39; Please delete a term "as". Unchanged, the expression ‘refer to as’ is correct
2. L226; Please delete a term "of". We moved the term “of” in front of the “( … )”, “in support of (…) and against …” L 230

3. L334; Replace a term "factor (single-noun)" by "factors (plural noun)".
4. L369-L370; This sentence is a repetition of the previous sentence. Please delete it.
5. L371; Please add a phrase just after the sentence like this. ... more likely triggers, which is similar to walk-run transition [1.6]. We added this at the start of the sentence (because of another addition suggested by reviewer#1). L373-375

6. L410; Please replace a term "muscle force" by "muscle activities".

Reviewer #3: I appreciate the major revisions made by the authors. I think they have made the manuscript stronger. The authors have appropriately resolved my minor comments. My major comment, i.e. that gravitational force along the skiing direction changes between incline conditions, and that the appropriate outcome variable therefore should be the sum of resistance forces rather than string tension, is discussed, although not at great depth. I agree with the authors argumentation on this point, although I would have preferred to present it differently. After all, the reported aim of the study was to test if power output at technique transition is constant across different speeds and inclines. Yet, the authors also address the question if “resistance force” is constant across different speeds and inclines. This is obviously a different hypothesis, and it is not straight-forward to answer if one is keen to distinguish between incline and “resistance force” (as should be clear from the present findings and discussion). The authors may consider again if they think the manuscript benefits from this discussion, or if it is better to keep it closer to the stated aim (i.e. power). Since I find nothing formally wrong in their arguments, this should be the authors’ prerogative.

The original (and current) aim was to investigate effect of power. However, because power was controlled by adding resistance fore, it made sense to us to also investigate the effect of this force on its own right, mostly to check and allow a better inference of our results. This reviewer suggested – and we agree – to also check for total resistance force. We prefer to keep our discussion to focus on the original main aim (power) but agree that the ‘force issue’ is of definite interest worth further investigation. We have added a few remarks to make it somewhat clearer that resistance force is a secondary (supporting) issue. L186-188. L378-380. L401-404

Minor comments:
- Line 111: missing word: “The comparison four such conditions…”

- Lines 214-219: Is imputing missing values in this way appropriate?

We believe so. Basically, we added the value for such ‘non-transition’ giving the least possible difference compared with other conditions. Any other value would reduce the power of the statistical test (i.e., rejecting H0 while it should not – see original comments revewer#2, new comment to reviewer#4) L219-222

- Figure 2 is difficult to understand, and (if I understand it right), it is not very important. Are you sure you want to initiate the results-section with this figure, and can it be presented/explained better?

We agree, indeed the figure is not critical for the paper and hard to understand. We reduced the complexity, and now use the same setup as Fig 5. Only depicting differences between max and min powers between ‘adjacent’ techniques. We explain in the legend that a positive value indicates overlap of powers for these techniques. We kept such figure and results in the text because these data ‘set the scene’ for the transition results.

- Lines 253-254 (or perhaps elsewhere, but this is where I thought about it): When interpreting effects of speed and incline, perhaps it’s worth reflecting on that the change in power output (at equivalent string force) between inclines was much bigger than the change in power output between speeds? By my calculations, power output during the two incline conditions differed by 44% while the speed conditions differed by 20%.

The reviewer is correct, be it that the differences are somewhat smaller. This has no effect on conclusions regarding power which is beyond reasonable doubt not a trigger, irrespective of what the exact default (=same resistance) power differences between conditions were.

We have added a small note in ‘methodological considerations’, concluding that one really cannot – based on our findings - argue (yet) for force being a trigger anyway. It now does make the reader explicitly aware of this difference. L461-468

- Lines 299-300: “i.e., the athletes retained a technique at greater resistive force when resistance was incremented versus decremented”. Do you mean power instead of force?

We see the point, in the hysteresis analysis power or force does not make a difference (comparison within one speed-incline condition). See also methods-statistics. We now make note of this explicitly here in the results. It seems a matter of taste in this choice: force that was used to initiate differences or power that was the goal variable to change. L302-303

- Lines 392-394: I’m not convinced by the statement “Still, the fact that additional force and total force (and power) at transition show opposite effects of incline (Fig. 4) suggests that these variables are essentially different.” Can you elaborate?

We have changed the final part of the sentence which we hope is clarifying. We added that clearly further study is required to elucidate this issue. L401-404

- Lines 394-396: Is the reference to Pellegrini et al. appropriate, given that they measured pole force while you measured (or estimated) total resistance force?

We believe so, because pole (propulsion) force and total resistance force are directly related by law of physics. We mention this explicitly now, but was already indicated (implicitly) in the next “increasing demand for propulsion” L406-407

- Lines 455-460: This seems unnecessary to mention in a conclusion.

We agree that this text is not a conclusion, but rather a rationale for some ‘warning’ about not excluding findings that are somewhat odd compared to most other findings. Still, we believe the information is noteworthy. We therefore created a new short section just before the ‘Methodological considerations’ that entails this entire last paragraph. L439-449 “Atypical behaviour”

This also makes the ‘concluding remarks’ closer to ‘plain conclusions’. [Please note that the section has “concluding remarks” as title, not “conclusions”.]

- Overall: Font size changes a few times in the manuscript body. Our apologies, now fixed.

Reviewer #4: The revision is not much improved, statistically, from the original submission. The results may still hold, but not in a statistically rigorous manner. As pointed out by a previous Reviewer, this is probably due to a lack of appropriate number of participants. The statistics and result sections are confusing. There is no design justification, power or sample size considerations motivating the small size of this investigation The test of normality for ANOVA is not adequately detailed. Reviewer 2, comment 4.1. is still relevant. The total of 14 is still much too inadequate considering the multiple endpoints and multiple comparison issues-especially with the p-value interpretation in the classical setting. Even for a Bayesian approach, the lack of sample size challenges the credibility and interpretation of the results.

We are somewhat confused about the comments raised here. We gave quite a detailed response including arguments for and against our approach to Rev. 2, including comment 4.1, which apparently was accepted by Rev2. In short, we agree with the principal issues raised both by Rev2 and Rev4. The relationship between N, p and effect size is well known. If the effect size is low, a large N is required to achieve a given low p. If effect size is large, a smaller N is required to achieve a given low p. In our paper, it is clear that for some outcome variables, effect size is large, as given by low p at a “low N”. This applies particularly for the main variable of interest, i.e., power at transition (note that resistance force is a secondary one): p=0.024, and 3x p<0.001.

In general, it is questionable if one means to state that N is independent of effect size: If one happens to arrive a p=0.0001 at N=10, it would likely be morally and ethically troublesome to collect N=100, just to have N=100 and p=0.000000001. We wish to emphasize this relationship: IF the true effect size in nature is large, and paper A arrives at p=0.0001 at a sample size of N=10, while paper B arrives at p=0.0001 at a sample size of N=100, then paper A has a larger effect size and ‘is more trustworthy’ than paper B; often people believe the paper with largest N is always better, but this is not the case. We performed a robustness check. L479-482

Regarding the normality assumption, it was stated in the text L.231-234 (now L237-239) that the assumption of normality was checked by inspection of QQ plots of residuals, as this is the core assumption. In Fig.1 of this document, for one variable, we show the results of the often-used Shapiro-Wilk test for normality for the dependent variable for each condition. If we were to follow the strict a=0.05 threshold, this variable is not proved to be non-normally distributed. Based on the QQ plots, however, we tend to see that the variable may deviate somewhat from a normal distribution. For the 2x2 RM ANOVA, the residuals are stored and the normality of these are shown to the right in the figure: they appear well within an acceptable range to state “approximately normally distributed”. The trend is very similar for all main outcome variables.

Figure 1. Normality assumption for an example variable. Top 4 panels show QQ plots of dependent variable for each condition (2 inclines, 2 speeds), table shows p-values for SW tests for each of these. Right panel shows QQ plots of residuals for the total 2x2 ANOVA model.

Regarding the “multiple endpoints” and the “multiple comparison”, the issue is not restricted to the p-value in the classical setting, but also an issue for the Bayes Factor. Our main outcome variables are external power and force, both which are mechanically linked/dependent on incline and speed, and to each other. This is one main reason for us taking a somewhat more ‘total, holistic’ approach, rather than interpreting each individual p-value (or bayes factor) independently and as separate findings (which is often done). Please note our previous reply about force being a secondary outcome variable, used to get a grasp on potential mechanisms guiding technique transitions.

It has possibly not been considered explicitly in the comments by reviewers that all tests are of the repeated form, strengthening statistical power.

The investigators note that they have a more holistic approach in presenting both the classical and Bayesian results (rather than solely focusing on ‘significance’) thus they can reduce the chance of committing type I errors. Again, one needs adequate sample size to reduce the chance of committing a type one error.
This holistic argument (classical and Bayesian) is not really justified. This confuses the results for the reader and one should use one or the other and present the results clearly.

Regarding our ‘holistic approach’ we must have been unclear in our writing: we did and do not at all mean to suggest that we are holistic just because we use both Bayesian and classical analysis. Our holistic approach refers in general to avoiding simpler interpretations of results based mainly/only on p-vales/Bayes factors (these are highly correlated, as Rev4 correctly states) above or below some threshold. Rather, we judge results and data more continuously, combining statistical outcomes, actual effect size (raw values) and data quality. We still believe that this more ‘holistic approach’ as well as using Bayes factors, better allow us to state something like “force may be a trigger” rather than “force is a trigger” just because p>0.05 (which would more likely happen if we restricted ourselves only to classical p-values, and judged results less holistically). We added a general reference [24] regarding this approach, indicating that this is not something we merely thought of ‘here and now’. L234

We believe that the reader will have no problems grasping the message.

The concept of missing value accommodation is not clear at all. The investigators note that (page 10 to 11) to allow them to use such ‘missing value’ in the statistical analysis, it was filled in with a fictitious value, one force or power step higher than the maximal value or one step less than the minimal value observed for that athlete during all four incline-speed settings. What is the reference for this approach?

Regarding the missing value accommodation issue: the data are not really missing (therefore the ‘ ’ denotation). The data points are present, i.e., the athletes behave regarding transitions (namely, the athletes do not transit as opposed to transiting at a given power/force), but there are no associated numbers. We made this now more explicit. There is no reference, we do not know of another study with this approach. Therefore the (elaborate) explanation, which reason (no number is not missing data) is amended L.219-222

Regarding the Bayesian approach, what underlying distributions are used for the prior constructs? How are the prior constructs arrived at? Presumably the approach is a Markov chain Markov modeling technique with diffuse or vague priors. All of the actual mechanics of this technique in this context is missing.

Regarding the prior settings for Bayesian approach, we thought this was explicitly written in the text. However, we see that it might have been not totally clear, so wording is slightly changed L.236-237. The use of a ‘default’ prior both for the ANOVA as well as for t-tests we see as fair regarding the uniform “prior” of the classical approach. Both for ANOVA as well as for t-tests, we ran JASPs robustness and sequential analysis tests for Bayes factors. Although Bayes factors would indeed change somewhat depending on prior and/or N, our conclusions would not be affected at all given different prior settings.
