# Supplementary material for: The role of external power demand on the choice of technique in classic cross-country skiing
Source: PLoS One. 2023 Mar 31;18(3):e0283794. doi: 10.1371/journal.pone.0283794 (PMC10065268; doi:10.1371/journal.pone.0283794)
Supplement: Attachment — Submitted filename: ReplyToComments03.docx [file pone.0283794.s004.docx]

**Authors’ replies to review Comments #03**

We thank the reviewers once more for their appraisal.

Reviewer #1: The authors have worked hard to improve the English writing/grammar.
I applaud their persistence. It was a pleasure to read the revised manuscript.
A few minor grammar items that remain can be easily fixed.

Line 99 too many negatives in one sentence. Simpler structure: “Still, in all these experiments changes in power were always associated with changes in incline and/or speed and so the isolated role of power was not clear.” Adopted, indeed a nicer way to put it. L98-100

Line 147-148 … assuring that they would be submaximal efforts, and the expectation…. (this fix avoids confusion between “load” and the hanging weights which might be called “loads”) Adopted L148

Line 187 … to investigate the role of power, it was also possible to elucidate the means by which power was altered, i.e. the resistive force. Adopted L188-189

Line 194 …external mechanical power was examined; any fluctuations in power due to the motion….. Adopted L195

Line 196 …associate losses were not considered. Adopted L196

Line 216 … conditions was regarded as evidence Adopted L216

Line 323 … but in opposite Adopted L323

Line 398 independent of the way Adopted L398

Lines 400 and 402 I’ve never heard this phrase ‘sense for propulsion’ but it would make more sense in English if it was ‘sense of propulsion’. More common would be ‘sense of effort’ Thank you for the suggestions. We have adopted ‘sense of effort’, which indeed sounds to us as more common. From the context it is clear that we here refer to effort in generating propulsive forces (not necessarily metabolic effort or alike). Thus, it need not be so explicit. L400 & 402

Line 458 i.e. smaller weight increments (reducing could be confused with when your experiment removed weight bags) Adopted L457

Line 463 I suggest you avoid two uses of the word “trigger”. Here, simply say: “This raises the question …” Adopted. Indeed, given the aim of study, a very good point L462

Line 467 “Still and anyway” is not something I’ve ever read or said. Instead, try: “In any case, the current findings….” Adopted. The use of such awkward expressions definitively gives away we’re not native English speakers. L466

Line 481 statistical Adopted L489

Line 481-482 I don’t understand what is meant here. Perhaps one of the other, more statistical reviewers can clarify. With the JASP software one can check, based on the present data set, the outcome when reducing N. Even though details may have changed, the inference would have been the same until reaching N as low as 7 or 8 (when the inference becomes more ‘undecided’). We rephrased slightly. “… until after N was reduced to 7 or 8.” L481-482

Reviewer #4: The authors did address the concerns for the holistic approach. However, it is still confusing to a reader expecting a consistent explanation for the statistical results.

We interpret this as the comment being basically that, i.e., a mere comment without expecting further changes in the manuscript on this issue. We understand that the reviewer still finds the analysis, and particularly its presentation, not optimal with regard to understanding for the readership, but not incorrect by itself. Although we appreciate and understand the comment (the analysis and inference are definitively not simple), we respectfully disagree that it is confusing, although it may to some. Both providing classic and Bayesian analysis may for others actually be enlightening. This is of course an issue for debate, but we hope it is not standing in the way for acceptance of this manuscript.
